# Association of HAMP Expression and CD8+ T‐Cell Infiltration With Atezolizumab–Bevacizumab Response in Hepatocellular Carcinoma

**DOI:** 10.1002/ags3.70158

**Published:** 2026-01-04

**Authors:** Shun Nakamura, Yuki Murakami, Yusuke Oshima, Takashi Masuda, Wataru Miyoshino, Hiroomi Takayama, Yoko Kawano, Teijiro Hirashita, Yuichi Endo, Masafumi Inomata

**Affiliations:** ^1^ Department of Gastroenterological and Pediatric Surgery, Faculty of Medicine Oita University Yufu Oita Japan; ^2^ Graduate School of Pharma‐Medical Sciences University of Toyama Toyama Toyama Japan; ^3^ Faculty of Engineering University of Toyama Toyama Toyama Japan; ^4^ Research Center for Pre‐Disease Science University of Toyama Toyama Toyama Japan

**Keywords:** atezolizumab, bevacizumab, hepatocellular carcinoma, hepcidin, RNA‐sequencing

## Abstract

**Aim:**

Atezolizumab combined with bevacizumab is the standard first‐line therapy for unresectable hepatocellular carcinoma; however, predictive biomarkers of therapeutic response remain undefined. We aimed to identify molecular features associated with therapeutic efficacy to develop personalized treatment strategies.

**Methods:**

Transcriptomic analyses were performed using public RNA‐sequencing datasets of patients with hepatocellular carcinoma receiving anti‐PD‐L1‐based therapy, comparing responders (complete response/partial response) with non‐responders (stable disease/progressive disease). Differentially expressed genes and enriched pathways were identified using integrated differential expression and pathway analysis. For validation, RNA‐sequencing was performed on institutional tumor samples (*n* = 6) underwent. Immunohistochemistry was performed on resected specimens (*n* = 9) to evaluate CD8+ tumor‐infiltrating lymphocytes and hepcidin protein expression encoded by the HAMP gene. Group comparisons for the pre‐specified immunohistochemistry endpoints were analyzed using exact Wilcoxon rank‐sum tests with multiplicity control (Holm adjustment).

**Results:**

Analysis of public datasets revealed distinct expression profiles in responders, enriched in immune‐related and chemokine signaling pathways. Candidate genes, including HAMP, TAT, and HRG, were upregulated in responders. In institutional samples, HAMP expression was significantly higher in preoperatively treated tumors (*p* = 0.001). Immunohistochemistry demonstrated greater CD8+ tumor‐infiltrating lymphocyte density (median 36.6 vs. 5.0 cells/high‐power field; exact Wilcoxon *p* = 0.032) and higher HAMP immunoreactive scores (median 4 vs. 0.5; *p* = 0.032) in responders than in non‐responders.

**Conclusions:**

Upregulation of HAMP and hepcidin protein expression, together with increased CD8+ T‐cell infiltration, was associated with a favorable response to atezolizumab–bevacizumab in patients with hepatocellular carcinoma. HAMP may serve as a component of a composite biomarker predictive of therapeutic sensitivity, warranting validation in larger, multi‐institutional cohorts.

## Graphical abstract

In hepatocellular carcinoma treated with atezolizumab–bevacizumab, responders showed RNA‐seq enrichment of immune and chemokine pathways with higher HAMP expression. In resected specimens, immunohistochemistry confirmed increased intratumoral CD8+ T‐cell density and hepcidin (HAMP), supporting HAMP plus CD8 as components of a composite predictor of treatment sensitivity.

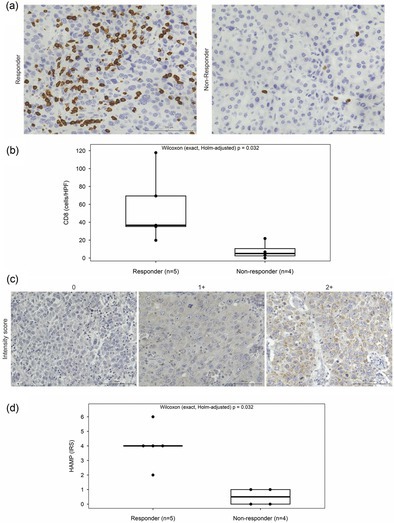

## 1 Introduction

Hepatocellular carcinoma (HCC) is a major global health challenge and one of the most common malignancies worldwide. In 2020, an estimated 905 677 new cases were diagnosed, ranking HCC as the sixth most frequent cancer. During the same period, 830 180 deaths were attributed to HCC, making it the third leading cause of cancer‐related mortality globally [1]. Surgical resection remains the mainstay of curative therapy for HCC, with pooled 5‐year overall survival (OS) of 56.2% and 5‐year recurrence‐free survival of 35.2% after curative resection, based on a global meta‐analysis of 110 studies [2]. HCC often progresses asymptomatically; thus, many patients are diagnosed at an advanced stage with unresectable, locally advanced, or metastatic disease. In such cases, surgical resection and locoregional therapies are not feasible, and systemic therapy is the primary treatment option.

Since its approval in 2007, sorafenib, a multikinase inhibitor, has been the standard first‐line systemic therapy for advanced HCC [3]. In 2018, lenvatinib was introduced as an alternative after the REFLECT trial established its non‐inferiority to sorafenib [4]. Despite these advances, both agents are limited by modest objective response rates and restricted survival benefits; achieving long‐term disease control remains difficult [5, 6]. Consequently, immunotherapy has emerged as a promising strategy. Atezolizumab (ATZ), an anti‐programmed death ligand 1 (PD‐L1) monoclonal antibody, combined with bevacizumab, an anti‐vascular endothelial growth factor monoclonal antibody, demonstrated superior outcomes in the IMbrave150 trial, a global phase III randomized controlled study published in 2020. Compared with sorafenib, the atezolizumab–bevacizumab (ATZ + BeV) regimen significantly improved OS and progression‐free survival in patients with unresectable HCC [7]. These findings led to the international approval of ATZ + BeV as the new first‐line standard of care for unresectable HCC.

Recent evidence indicates that the tumor immune microenvironment critically influences the efficacy of ATZ + BeV therapy. Responders have been shown to display increased CD8+ T‐cell infiltration and elevated expression of pro‐inflammatory chemokines such as CXCL9 and CXCL10, alongside activation of immune‐related pathways including antigen presentation and interferon signaling [8, 9, 10, 11]. Despite these insights, there is substantial interindividual variability in treatment, and no validated predictive biomarkers are currently available. Therefore, the present study aimed to investigate potential molecular determinants of response to ATZ + BeV therapy in HCC, with the ultimate goal of identifying predictive biomarkers that could support the development of personalized treatment strategies.

## 2 Materials and Methods

### 2.1 Collection and Analysis of Public RNA‐Seq Datasets

We analyzed publicly available RNA‐Seq data from the Gene Expression Omnibus (GEO) database (https://www.ncbi.nlm.nih.gov/geo/). Specifically, we retrieved GSE279750, which comprises RNA‐Seq data from HCC tumor samples obtained from 10 patients who underwent anti‐PD‐L1‐based therapy [12]. The cohort comprised six responders and four non‐responders, categorized according to radiographic treatment response: patients achieving a complete or partial response were defined as the responder group, while those with stable or progressive disease were defined as the non‐responder group.

Differential expression analysis was conducted using integrated differential expression and pathway analysis [13, 14]. Differentially expressed genes (DEGs) were identified between responders and non‐responders using a false discovery rate (FDR)‐adjusted *p*‐value of < 0.05 and absolute log_2_ fold change of ≥ 1.5 as thresholds. Functional enrichment analysis of Gene Ontology (GO) terms, including biological processes, cellular components, and molecular functions, was then performed to assess the differences between groups. Principal component analysis (PCA) of normalized RNA‐Seq data was used to visualize global transcriptional variation, with principal component scores plotted to evaluate similarities and differences in expression profiles between the responder and non‐responder groups.

### 2.2 Institutional Sample Collection and RNA‐Seq Analysis

Between 2020 and 2023, frozen tumor specimens were collected from six patients with pathologically confirmed HCC who underwent hepatectomy at our institution. Among them, three patients received preoperative combination therapy with ATZ + BeV (designated Atz_Human1–3), all of whom exhibited pathological tumor regression (pathological treatment effect grade ≥ 2 [TE ≥ 2]) and subsequently underwent conversion surgery. The remaining three underwent surgery without prior systemic therapy (none_Human1–3). Clinicopathologic characteristics, preoperative therapy details, and washout periods are summarized in Table 1.

**TABLE 1:** Clinicopathologic characteristics of the institutional cohort.

Case	Age (years)	Sex	Etiology	Cirrhosis	BCLC stage	Tumor size (mm)	Number of tumors	Preoperative therapy	Washout period (days)
Atz_Human1	81	M	NBNC	No	C	30	5	TACE × 3; Lenvatinib; ATZ + BeV × 6	31
Atz_Human2	65	M	NBNC	Yes	C	60	1	ATZ + BeV × 5	23
Atz_Human3	84	M	HBV	No	C	70	1	ATZ + BeV × 5	24
none_Human1	89	M	NBNC	Yes	B	40	2	None	N/A
none_Human2	70	M	HCV	No	A	50	1	None	N/A
none_Human3	76	M	NBNC	No	B	35	2	None	N/A

*Note:* Washout period: days from the last preoperative therapy to surgery, N/A for untreated cases.

Abbreviations: ATZ, atezolizumab; BCLC, Barcelona Clinic Liver Cancer; BeV, bevacizumab; HBV, hepatitis B virus; HCV, hepatitis C virus; NBNC, non‐B non‐C; TACE, transarterial chemoembolization.

Total RNA was extracted using the RNeasy Mini Kit (QIAGEN, Hilden, Germany). RNA quality control, library preparation, and sequencing were outsourced to Rhelixa Inc. (Tokyo, Japan). Libraries were prepared using the NEBNext Poly(A) mRNA Magnetic Isolation Module (E7490; New England Biolabs, Ipswich, MA, USA) and the NEBNext Ultra II Directional RNA Library Prep Kit (E7760; New England Biolabs), followed by sequencing on the Illumina NovaSeq X Plus platform to generate 150‐bp paired‐end reads. Each sample yielded approximately 40 million reads (20 million pairs), corresponding to approximately 6 GB of sequence data. DEG analysis, GO functional enrichment, and PCA were performed using the same analytical pipeline applied to the public dataset [13, 14], thereby ensuring methodological consistency. In the institutional RNA‐Seq cohort, we compared post‐ATZ + BeV resection specimens with untreated surgical controls which are not baseline responder/non‐responder samples. Accordingly, the institutional RNA‐Seq should be interpreted as an exploratory analysis of treatment‐exposed versus untreated tumors rather than a predictive comparison. One treated sample failed RNA QC, yielding *n* = 2 treated versus *n* = 3 untreated for downstream analyses. Given the limited institutional sample size and the treated–untreated design, we prespecified a triangulation strategy to assess transcriptomic robustness: (i) check cross‐dataset directionality of HAMP (public responders vs. non‐responders; institutional treated vs. untreated) and (ii) verify concordance between RNA‐Seq signals and protein‐level IHC; public dataset pathway enrichments served as an external biological check.

### 2.3 Immunohistochemistry

Formalin‐fixed, paraffin‐embedded tumor specimens were obtained postoperatively from nine patients with HCC who underwent surgical resection following preoperative ATZ + BeV therapy at our institution. Patients achieving partial response were classified as responders, whereas those with stable or progressive disease were classified as non‐responders. In total, five patients were classified as responders and four as non‐responders. CD8 immunostaining was performed using an automated immunostainer (Leica BOND) with a mouse monoclonal anti‐CD8 antibody (clone 4B11; Leica Biosystems, catalog no. PA0183), whereas HAMP immunostaining was performed manually using a rabbit polyclonal anti‐HAMP antibody (ab30760; Abcam, dilution 1:100). In both protocols, HRP‐labeled polymer secondary antibodies (Histofine Simple Stain MAX PO; Nichirei Biosciences) were applied, signals were visualized using 3,3‐diaminobenzidine (DAB), and nuclei were counterstained with hematoxylin.

The indication for preoperative ATZ + BeV was advanced tumor burden with macrovascular invasion (Vp ≥ 3, B ≥ 3, Vv ≥ 2) and/or nodal or distant metastasis. Liver function was preferably Child–Pugh A; selected Child–Pugh B cases were included when clinically acceptable. Prior locoregional/systemic therapies underwent a protocolized washout; the final preoperative cycle omitted bevacizumab, and surgery was scheduled at least 6 weeks after the last bevacizumab dose and at least 3 weeks after the last atezolizumab dose. These criteria were uniformly applied across the cohorts, and potential selection bias is acknowledged.

Evaluation of CD8+ tumor‐infiltrating lymphocytes (TILs) was conducted by selecting five high‐power fields (HPFs, ×400) with the highest infiltration density, as described previously [15]. The mean number of positively stained cells per HPF was calculated for each case. HAMP expression was quantified using the immunoreactive score (IRS) method [15, 16], based on both the proportion of positively stained tumor cells (0 = 0%, 1 = < 10%, 2 = 10%–50%, 3 = 50%–80%, and 4 = > 80%) and staining intensity (0 = none, 1 = weak, 2 = moderate, 3 = strong). The final IRS was obtained by multiplying the percentage and intensity scores, yielding a range of 0–12.

All immunohistochemical (IHC) assessments were independently performed by two board‐certified pathologists who were blinded to clinical outcomes and discordant evaluations were resolved by consensus. To minimize measurement bias, the slides were randomized. Hotspot selection followed pre‐specified exclusion zones (necrosis, edges, portal tracts); staining batches were balanced across groups with internal controls and image capture used fixed magnification and exposure settings. The clinicopathologic characteristics of the nine patients are summarized in Table 2.

**TABLE 2:** Baseline clinicopathologic characteristics of the IHC cohort stratified by radiologic response to preoperative atezolizumab plus bevacizumab (ATZ + BeV).

	Responder (*n* = 5)	Non‐responder (*n* = 4)	*p*
Median age (years, IQR)	74 (69–84)	72.5 (65.75–77.25)	0.621
Sex, *n* (%) (male/female)	5 (100%)/0 (0%)	3 (75%)/1 (25%)	0.444
Etiology (HBV/HCV/NBNC)	2 (40%)/0 (0%)/3 (60%)	2 (50%)/1 (25%)/1 (25%)	0.714
Child‐Pugh (A/B/C)	4 (80%)/1 (20%)/0 (0%)	3 (75%)/1 (25%)/0 (0%)	1.000
Cirrhosis (−/+)	3 (60%)/2 (40%)	1 (25%)/3 (75%)	0.524
BCLC stage (A/B/C)	0 (0%)/0 (0%)/5 (100%)	0 (0%)/0 (0%)/4 (100%)	NA
Median tumor size (mm)	100 (80–120)	30 (27–32.5)	0.019
Number of tumors (1/≥ 2)	3 (60%)/2 (40%)	2 (50%)/2 (50%)	1.000
Median AFP level (ng/mL, IQR)	6619 (1683–25 667)	8.81 (2.58–30.17)	0.020
Median PIVKA‐II (mAU/mL, IQR)	29 756 (6618–75 000)	173.85 (29.5–743.25)	0.037

Abbreviations: AFP, alpha‐fetoprotein; ATZ, atezolizumab; BCLC, Barcelona Clinic Liver Cancer; BeV, bevacizumab; HBV, hepatitis B virus; HCV, hepatitis C virus; IQR, interquartile range; NBNC, non‐B non‐C; PIVKA‐II, protein induced by vitamin K absence or antagonist‐II (des‐γ‐carboxy prothrombin).

### 2.4 Statistical Analysis

Categorical variables are summarized as counts (percentages) and were compared using Fisher's exact test. Continuous variables are summarized as medians (IQRs) and were compared using the exact Wilcoxon rank‐sum test; mean ± SD values are additionally reported when informative. All tests were two‐sided with *α* = 0.05. *p*‐values for baseline characteristics are considered exploratory and were not adjusted for multiple comparisons. Analyses used available cases; missing data, if any, are indicated in the tables.

Two pre‐specified IHC endpoints (CD8+ TIL density and HAMP IRS) were compared using the exact two‐sided Wilcoxon rank‐sum test. For CD8+ TIL density, the patient‐level value represented the mean value across five pre‐specified HPFs. For HAMP, an IRS (intensity 0–3 × proportion 0–4; range, 0–12) was assigned at the individual case level, without any per‐HPF averaging. Effect sizes were calculated using the Hodges–Lehmann median difference and Cliff's delta, along with 95% BCa bootstrap CIs (≥ 20 000 resamples; fixed seed). Multiplicity across the two primary comparisons was controlled with Holm adjustment, and adjusted *p*‐values are reported. In cases where closed‐form exact *p*‐values were unavailable (e.g., ties), permutation‐based exact *p*‐values were used and found to be concordant. Analyses and figures were produced using R software version 4.4.2 (2024‐10‐31), “Pile of Leaves.”

To address baseline imbalances (tumor size, AFP, PIVKA‐II; Table 2), we used Firth logistic regression with response as the outcome and either HAMP IRS or CD8 density as the predictor, adjusted singly for each covariate; we also performed size‐stratified exact tests (Mantel–Haenszel common odds ratio; two‐sided *α* = 0.05). *IHC sensitivity*: CD8 comparisons were recomputed using the HPF median (vs. mean) but the results were consistent. Exploratory prediction: a composite logistic score was built from *z*‐scored HAMP IRS + CD8 density. Performance was summarized by apparent AUC with percentile 95% CIs from 2000 bootstraps, with ROC plots; decision‐curve analysis evaluated net benefit across 10%–40% thresholds versus treat‐all/none and single‐marker models. Given that *n* = 9, these analyses are descriptive, without optimism correction or external validation.

## 3 Results

### 3.1 Differential Gene Expression Analysis of Public RNA‐Seq Data

Transcriptomic comparisons between the responder (R1–R6) and non‐responder (NR1–NR4) groups (Figure S1) demonstrated clear intergroup differences. Heatmap visualization of the top 2000 DEGs (Figure S1a) revealed a distinct gene cluster characterized by high expression in responders and low expression in non‐responders. MA and volcano plots (Figure S1b,c) further highlighted statistically significant DEGs, represented as red (upregulated) or green (downregulated) data points. These findings indicate that specific transcriptional alterations are associated with responsiveness to ATZ + BeV therapy. The direction of HAMP upregulation in responders in the public dataset was concordant with the institutional treated cohort and IHC findings, supporting cross‐dataset triangulation.

### 3.2 Pathway Enrichment Analysis of Public RNA‐Seq Data

**FIGURE 1:**
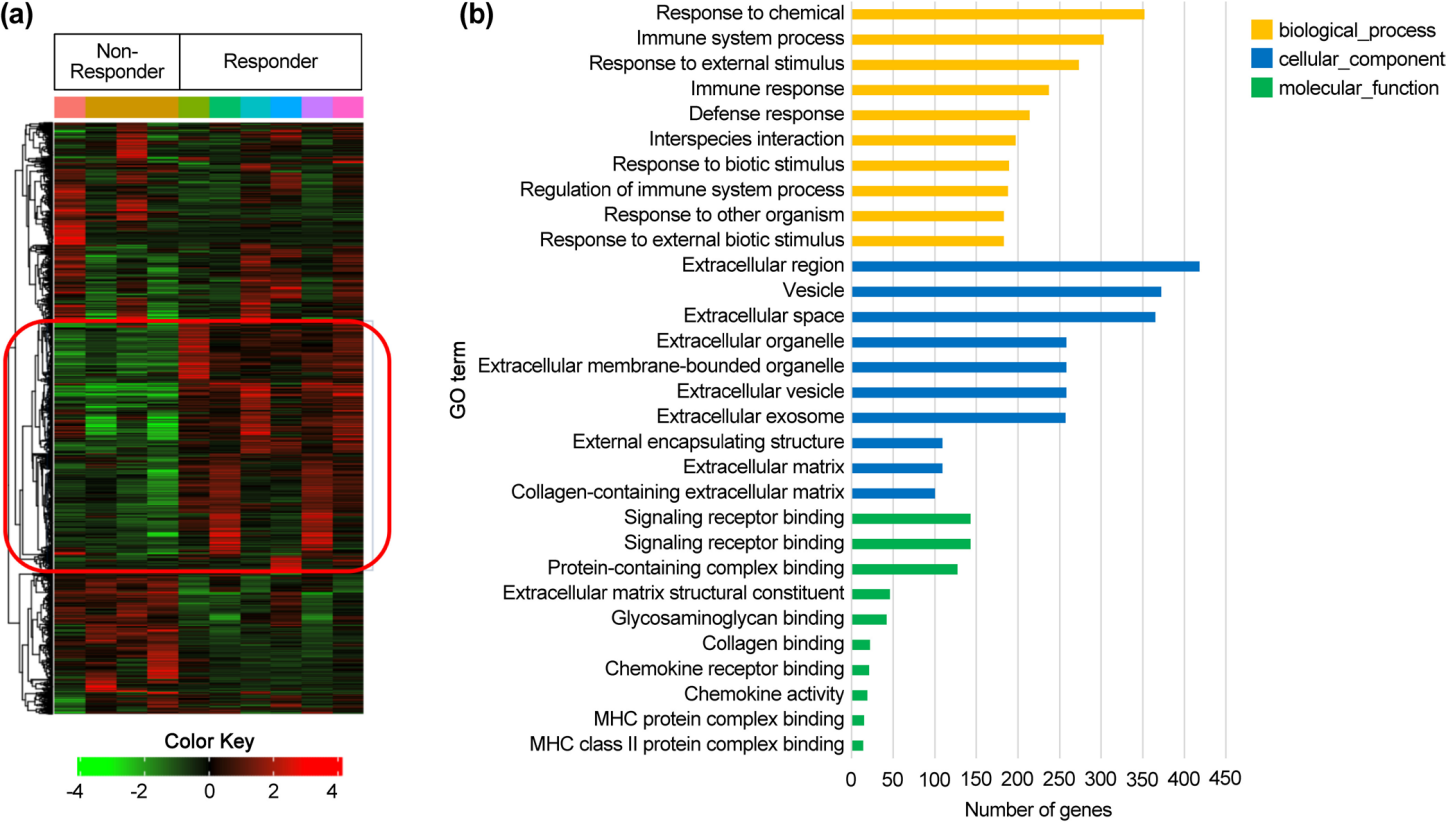
(a) Heatmap of genes upregulated in responders compared with non‐responders in public RNA‐sequencing data. (b) Gene Ontology (GO) pathway enrichment of upregulated genes, highlighting immune‐related and chemokine signaling pathways. The GO term “Biological process involved in interspecies interaction between organisms (GO:0044419)” is abbreviated as “interspecies interaction” for readability.

Genes upregulated in responders, highlighted by the red box in the heatmap (Figure 1a), were subjected to functional enrichment analysis. The results revealed significant enrichment of immune‐related pathways, extracellular matrix organization, and chemokine signaling (Figure 1b). These enriched pathways suggest that genes overexpressed in responders may contribute to the modulation of the tumor immune microenvironment, thereby enhancing the therapeutic efficacy of ATZ + BeV. These immune/chemokine enrichments served as an external biological check concordant with the institutional observations.

### 3.3 Principal Component Analysis of Gene Expression Profiles Based on Public RNA‐Seq Data

**FIGURE 2:**
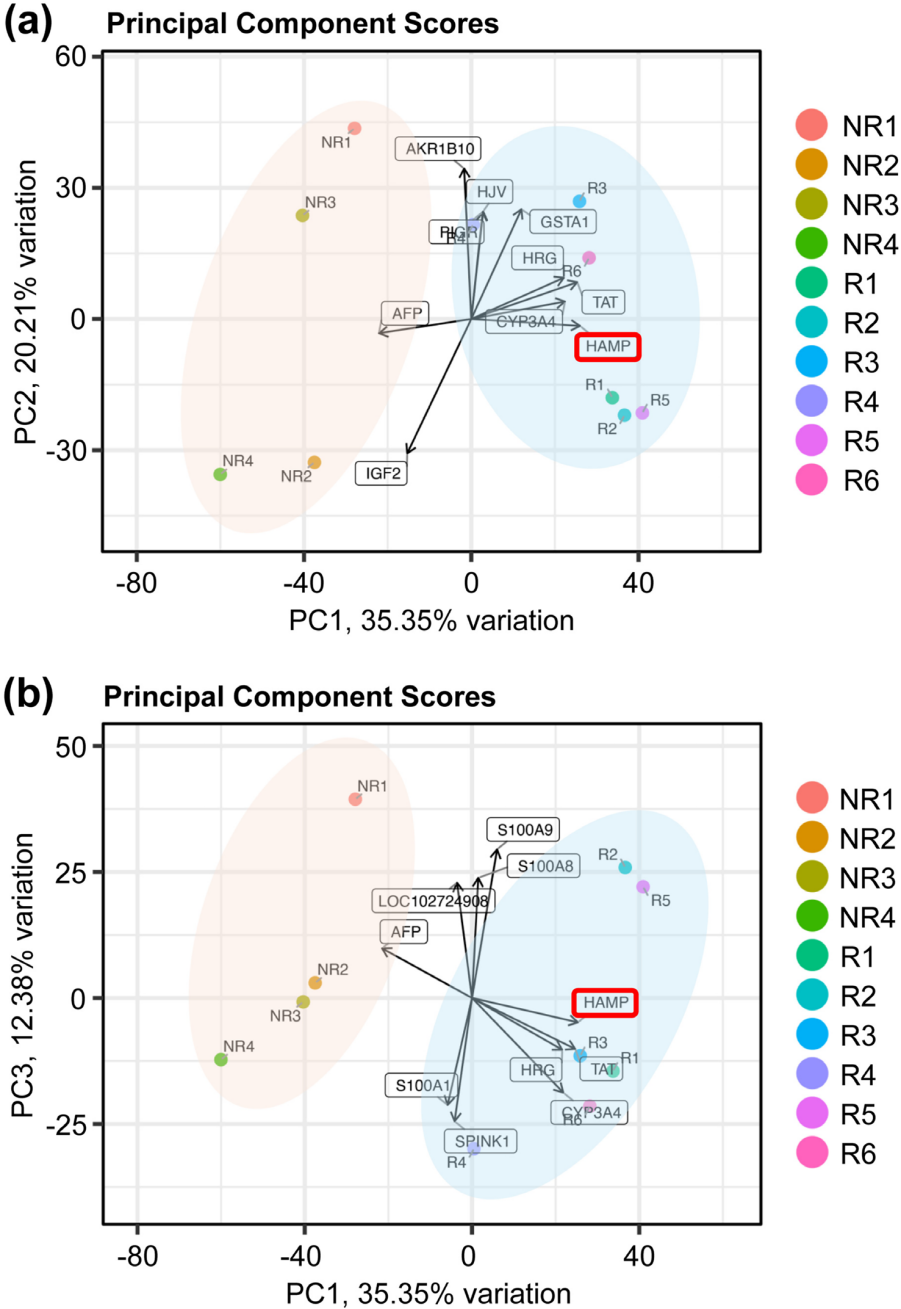
Principal component analysis (PCA) of public RNA‐sequencing transcriptional profiles. PC1 versus PC2 and PC1 versus PC3 plots show separation between responder and non‐responder groups; vectors toward the responder cluster include HAMP, TAT, and HRG.

PCA further confirmed the differences in gene expression profiles between responders and non‐responders (Figure 2). PC1 and PC2 accounted for approximately 40% and 25% of the total variance, respectively, with the two groups showing distinct clustering (Figure 2a). A comparable separation was observed in the PC1 versus PC3 plot (Figure 2b). Notably, vectors oriented toward the responder group (R1–R6) were associated with genes such as HAMP, TAT, and HRG, implicating these genes as contributors to the unique expression signatures observed in treatment responders.

### 3.4 Differential Gene Expression Analysis of Institutional RNA‐Seq Data

Transcriptomic profiling of the institutional HCC tumor samples revealed similar trends as described above. Due to poor RNA quality, Atz_Human2 was excluded from downstream DEG and pathway enrichment analyses. The results are summarized in Figure S2. Heatmap visualization (Figure S2a) demonstrated distinct expression patterns between treated and untreated tumors. MA and volcano plots (Figure S2b,c) identified significant DEGs, with upregulated genes marked in red and downregulated genes in green. These results are consistent with therapy‐associated transcriptional reprogramming in preoperatively treated tumors, which may explain the enhanced therapeutic response observed in these patients.

### 3.5 Pathway Enrichment Analysis of Institutional RNA‐Seq Data

**FIGURE 3:**
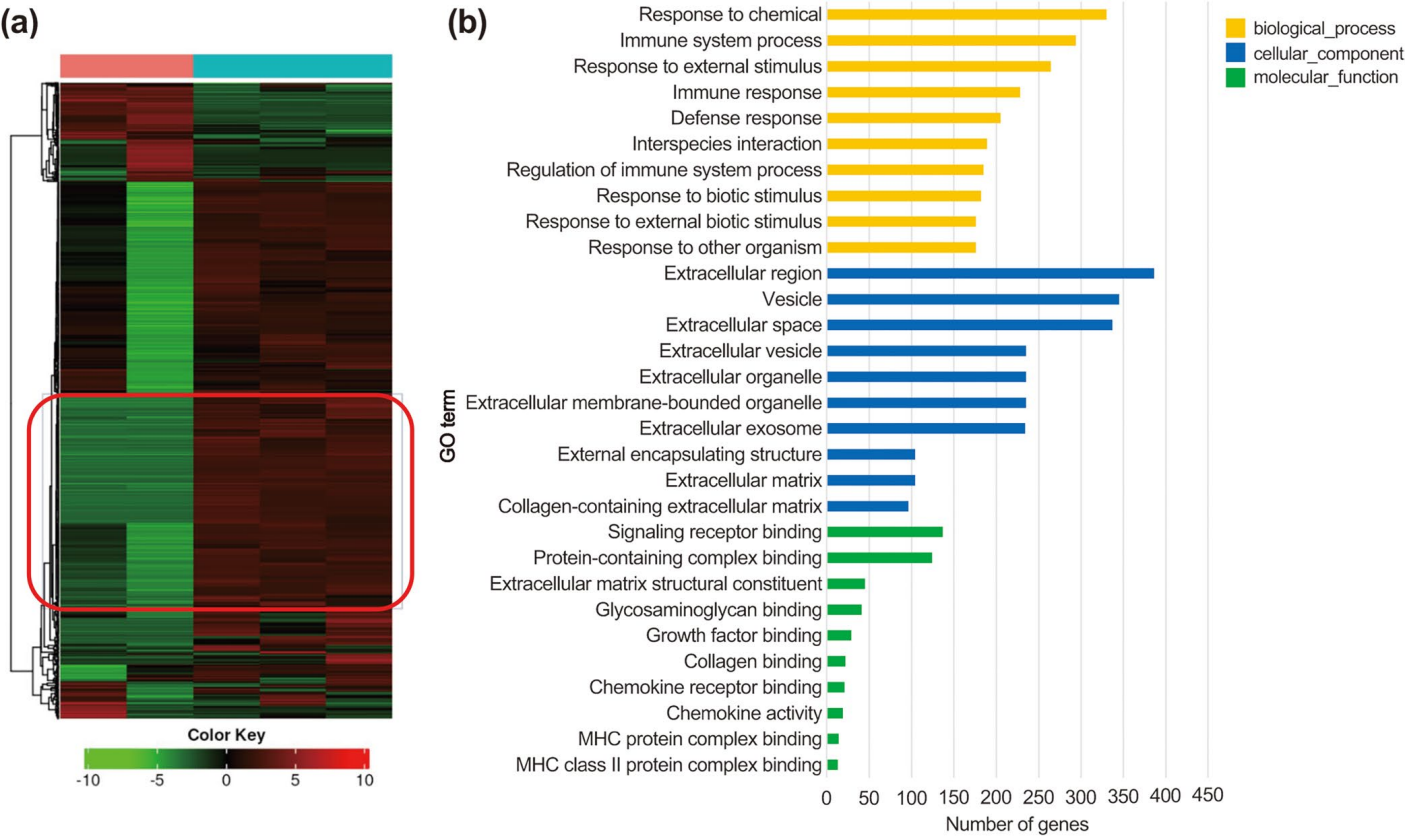
(a) Cluster of genes (red border) specifically upregulated in the institutional treatment group (atezolizumab plus bevacizumab, ATZ + BeV) versus untreated controls. (b) Pathway enrichment of these genes, emphasizing immune responses and tumor microenvironment modulation. The GO term “Biological process involved in interspecies interaction between organisms (GO:0044419)” is abbreviated as “interspecies interaction” for readability.

Pathway enrichment analysis was performed on genes specifically upregulated in the treatment group (Figure 3). A cluster of genes shown in the heatmap (Figure 3a) showed high expression in the treated samples but low expression in the untreated controls. Enrichment analysis (Figure 3b) identified multiple significant pathways, with many linked to immune responses and the modulation of the tumor microenvironment (TME). These results suggest that ATZ + BeV therapy induces transcriptional reprogramming that enhances immune activation within the TME, thereby contributing to its therapeutic effect.

### 3.6 Principal Component and Individual Gene Expression Analyses of Institutional RNA‐Seq Data

**FIGURE 4:**
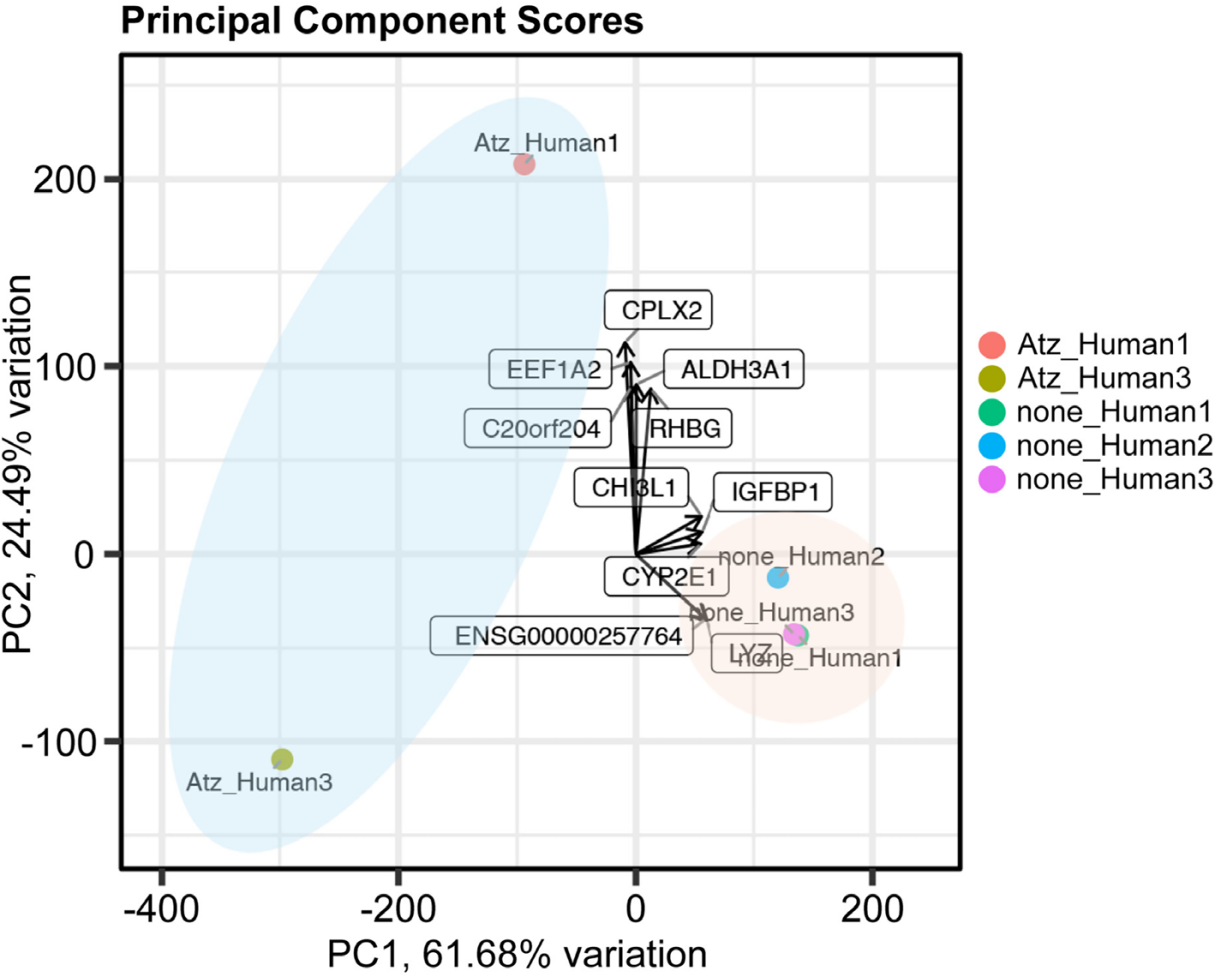
PCA of institutional RNA‐sequencing data comparing treated and untreated samples. A clear group separation was observed although the contributing genes differed from those in the public dataset.

PCA of the institutional dataset revealed a clear separation between treatment and control groups (Figure 4). However, the genes contributing to this separation differed from those identified in the public dataset. In contrast, individual gene‐level analysis demonstrated significantly higher HAMP expression in the treatment group (*p* = 0.001). Although PCA did not reveal overlapping driver genes with the public dataset, the consistent upregulation of HAMP suggests it may serve as a candidate biomarker associated with responsiveness to ATZ + BeV therapy.

### 3.7 Immunohistochemical Analysis of Resected Specimens

**FIGURE 5:**
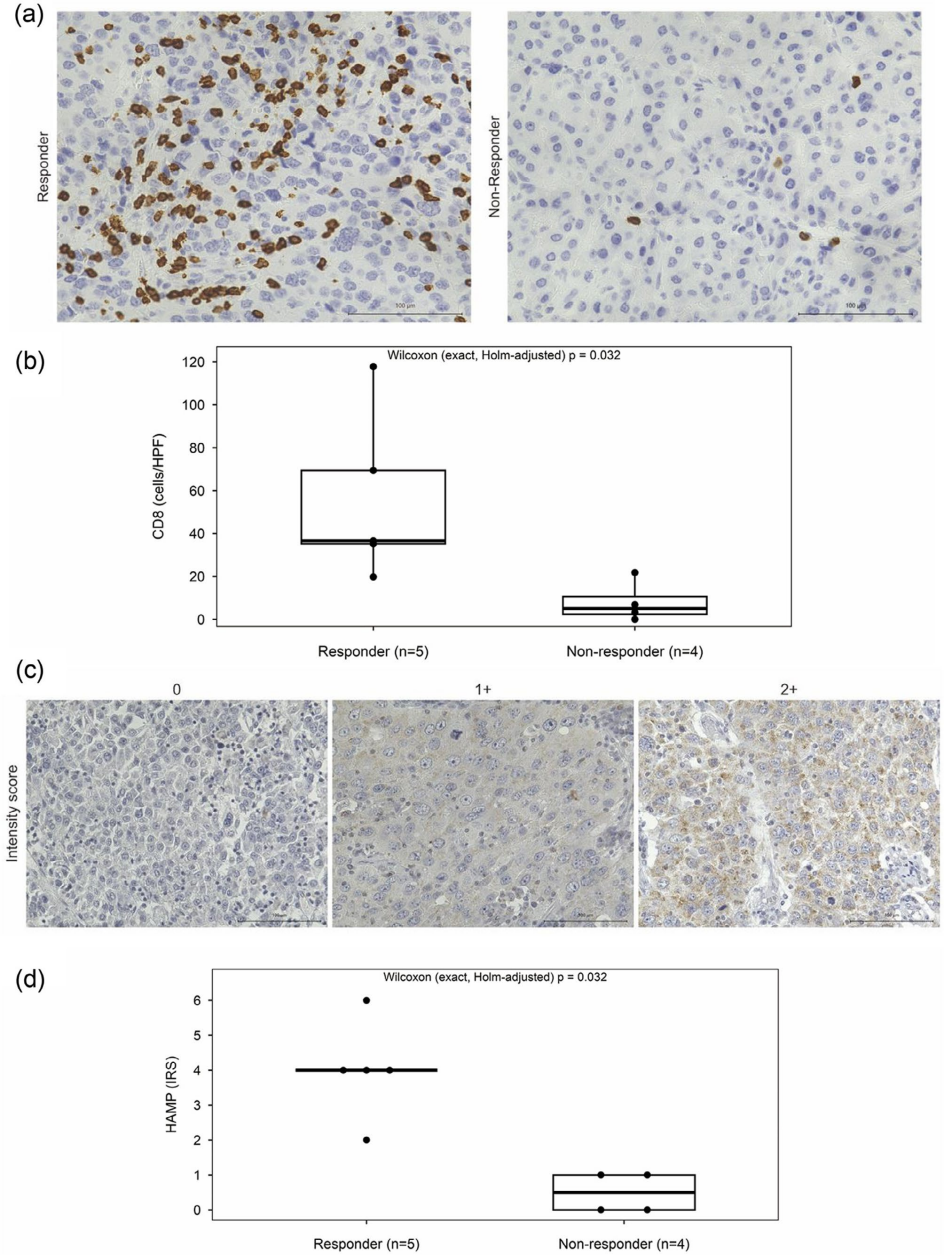
(a, b) CD8 immunohistochemistry (IHC). (c, d) HAMP (hepcidin) IHC. (a) Representative IHC images from responder (partial response) and non‐responder (stable disease/progressive disease) cases (scale bar = 100 μm). (b) Quantification of CD8+ tumor‐infiltrating lymphocytes (TILs), showing significantly higher density in responders versus non‐responders (median 36.6 vs. 5.0 cells/high‐power field [HPF]; exact Wilcoxon rank‐sum, Holm‐adjusted *p* = 0.032). Effect sizes: Hodges–Lehmann median difference 34.3 cells/HPF (95% CI, 14.8–96.0); Cliff's delta 0.90 (95% CI, 0.50–1.00). (c) Representative images corresponding to intensity scores of 2+, 1+, and 0 (scale bar = 100 μm). (d) Immunoreactive score (IRS) comparison showing significantly higher HAMP expression in responders versus non‐responders (median = 4 [IQR = 4–4] vs. 0.5 [0–1]; exact Wilcoxon rank‐sum, exact Holm‐adjusted *p* = 0.032). Effect sizes: Hodges–Lehmann median difference 3.5 points (95% CI, 2.0–5.0); Cliff's delta 1.00 (95% CI, 1.00–1.00).

Among the nine analyzed cases (Table 2), five were classified as responders and four as non‐responders based on the radiographic treatment evaluation. IHC staining for CD8 demonstrated significantly higher densities of CD8+ TILs in the responder group compared with those in the non‐responder group (median 36.6 vs. 5.0 cells/HPF; exact Wilcoxon rank‐sum [Holm‐adjusted *p* = 0.032]; Hodges–Lehmann difference 34.3 cells/HPF [95% CI, 14.8–96.0]; Cliff's delta 0.90 [95% CI, 0.50–1.00]; Figure 5). Representative IHC images are shown in Figure 5a, with quantitative comparisons in Figure 5b.

HAMP (hepcidin) expression, evaluated based on the IRS, was higher in the responders than in the non‐responders (median, 4 [IQR 4–4] vs. 0.5 [0–1]); the two‐sided exact permutation Wilcoxon test remained significant after multiplicity adjustment (Holm‐adjusted *p* = 0.032, adjusted for the two pre‐specified IHC endpoints). The Hodges–Lehmann median difference was 3.5 points (95% CI, 2.0–5.0), and Cliff's delta was 1.00 (95% CI, 1.00–1.00; Figure 5). Representative IHC images corresponding to staining intensities of 2+, 1+, and 0 are shown in Figure 5c, with quantitative results presented in Figure 5d.

**FIGURE 6:**
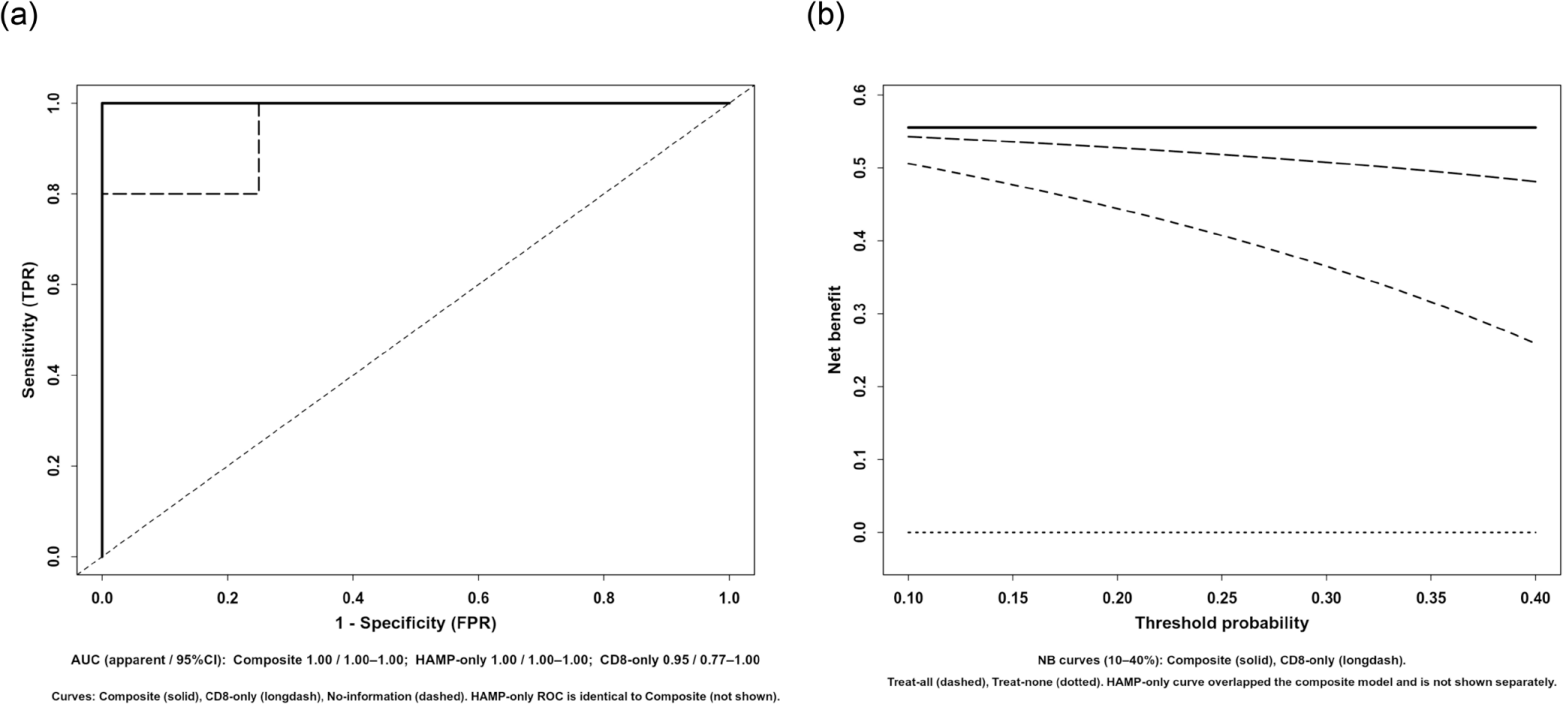
(a) ROC curves for the composite logistic model combining HAMP and CD8 versus single‐marker models. Apparent AUC with bootstrap 95% CI: Composite 1.00 (1.00–1.00); HAMP‐only 1.00 (1.00–1.00); CD8‐only 0.95 (0.77–1.00); *n* = 9. Values reflect apparent performance without optimism correction or external validation. (b) Decision‐curve analysis across threshold probabilities 10%–40%. The composite and HAMP‐only models both show positive net benefit versus treat‐all, treat‐none, and the CD8‐only model over this range; the HAMP‐only curve overlapped the composite model and is therefore not shown separately. Findings are based on *n* = 9, are exploratory and intended for hypothesis generation.

A median‐based sensitivity analysis of CD8 across the five HPFs yielded qualitatively unchanged group differences, supporting robustness of the IHC quantification. Adjusted associations remained directionally consistent after size/AFP/PIVKA‐II adjustment, and size‐stratified exact tests with the Mantel–Haenszel estimate were concordant. In exploratory composite‐score modeling, the HAMP+CD8 model showed higher apparent discrimination than either marker alone by ROC, and decision‐curve analysis indicated positive net benefit over “treat‐all/none” across 10%–40% threshold probabilities (Figure 6a,b). Given the small sample size, these findings are descriptive and not intended as a validated prediction.

## 4 Discussion

In this study, enrichment analysis indicated that activation of tumor immune responses and chemokine‐related pathways was associated with responsiveness to ATZ + BeV therapy in patients with HCC. Consistent with recent reports, responder tumors exhibited features of a T cell‐inflamed microenvironment, including higher CD8+ infiltration, effector T‐cell signatures, and enhanced chemokine signaling (e.g., CXCL9/10) [8, 9, 10, 11, 17, 18]. Notably, HAMP expression distinguished responders from non‐responders, with IHC analysis confirming significantly higher intratumoral CD8+ T‐cell density and HAMP IRS in responders. These findings suggest that HAMP contributes to an inflamed TME and may serve as part of a composite biomarker panel for patient stratification. Pretreatment biopsies are uncommon in HCC; thus, our post‐treatment resection data support association rather than prediction, and we cannot determine whether high HAMP was present at baseline or induced by ATZ + BeV [7]. Hepcidin‐25, the blood form of HAMP, can be quantified by validated LC–MS/MS [19]; however, circulating hepcidin or HAMP has not been prospectively validated to predict ATZ + BeV response. Any exploratory serum analysis should adjust for iron status, systemic inflammation, and renal function, and would require prospective evaluation. If pretreatment serum is available, correlating baseline hepcidin‐25 with radiographic or pathologic response could be considered.

HAMP encodes hepcidin, a peptide hormone predominantly produced in the liver that acts as the master regulator of systemic iron homeostasis. Hepcidin regulates iron efflux by interacting with ferroportin and inducing its internalization and degradation, thereby limiting extracellular iron release and maintaining systemic iron balance [20, 21]. Previous studies, including analyses of The Cancer Genome Atlas, have shown that HAMP expression is frequently downregulated in HCC compared with normal liver tissue. Such downregulation may promote excessive iron accumulation in hepatocytes and within the TME, contributing to tumor progression through oxidative stress and sustained activation of STAT3 signaling. Suppression of HAMP has also been linked to enhanced malignancy via the cyclin‐dependent kinase 1 (CDK1)/STAT3 pathway, suggesting that aberrant iron metabolism may represent a mechanistic axis of HCC progression [22, 23]. Beyond tumor growth, alterations in iron homeostasis may modulate immune responses by influencing macrophage polarization, T‐cell activity, and reactive oxygen species production, although context‐dependent effects have been reported [22, 24, 25]. Thus, our results support viewing HAMP within the broader interplay of iron regulation and immune activity rather than as an isolated driver.

Bevacizumab may further enhance immune checkpoint inhibitor activity through vascular normalization, which can reduce hypoxia, restore endothelial function, and facilitate lymphocyte trafficking [26, 27]. Together, a HAMP‐associated inflamed TME and vascular normalization provide a biologically coherent explanation for the therapeutic efficacy of ATZ + BeV [7, 9], although causal relationships require further validation.

Recent studies suggest that HAMP influences not only iron metabolism but also the tumor immune microenvironment [22, 25, 28]. Excessive iron accumulation increases oxidative stress and can modulate macrophage polarization in a context‐dependent manner (e.g., promoting immunosuppressive programs under specific conditions [24, 25]). Transcriptomic analyses further indicate that HAMP expression positively correlates with the infiltration of regulatory T cells, macrophages, and CD8+ T cells, implicating iron regulation in shaping immune cell dynamics. Elevated HAMP expression may promote ferroportin degradation, reducing local iron overload and thereby alleviating immunosuppressive conditions and fostering an immune‐activated state [20, 21]. Conversely, inflammatory cytokines within the TME, including IFN‐γ, can induce PD‐L1 expression on tumor cells via JAK1/2–STAT and PI3K/AKT signaling, facilitating immune evasion [17, 18, 29]. These pathways may act synergistically with STAT3 activation driven by iron dysregulation [21], reinforcing immune suppression and potentially reducing the efficacy of immune checkpoint blockade. Thus, reduced HAMP expression may not only promote tumor progression but also contribute to treatment resistance by creating an immunosuppressive TME and enhancing PD‐L1 expression.

In our cohort, elevated HAMP expression in responders was interpreted as part of an inflamed TME signature rather than a direct driver of PD‐L1 expression or CD8+ T‐cell infiltration. PD‐L1 elevation is more likely an adaptive response to IFN‐γ within a T cell‐inflamed milieu, which remains compatible with the clinical benefits of ATZ + BeV therapy [7, 9]. Accordingly, we do not assume a direct HAMP → STAT3 → PD‐L1 causal pathway but interpret the relationship as an association [17, 18, 29]. Our institutional RNA‐Seq compared treated resection specimens with untreated surgical controls; thus, these results contextualize treatment‐associated transcriptional differences rather than baseline predictive signals. At the protein level, we confirmed higher HAMP in radiographically defined responders than that in non‐responders using prespecified IHC endpoints with multiplicity control. Despite the small sample size and differing comparison frames across cohorts, the direction of HAMP change was consistent across the datasets, supporting HAMP as part of a composite immune marker rather than a stand‐alone predictor. Differences in gene lists likely reflect the different comparison frames, pre‐ versus post‐treatment sampling time points, analytic pipelines, and case mix.

In line with these findings, multiple landmark studies have shown that the clinical efficacy of ATZ + BeV is strongly influenced by the preexisting immune landscape of HCC [7, 8, 9, 10, 11, 28]. Lim et al. reported that responders exhibited activation of CD8+ T cells, tumor‐associated macrophages, and dendritic cells, accompanied by increased secretion of chemokines such as CXCL9 and CXCL10, which recruit CXCR3+ cytotoxic T cells into the TME [8]. Similarly, Cappuyns et al. demonstrated that CXCL10^+^ macrophages and clonal expansion of effector CD8+ T cells defined a responsive tumor state [11]. Large‐scale analyses further identified two therapeutic phenotypes—immune‐dominant and angiogenesis‐dominant—underscoring the central role of immune activation in therapeutic outcomes [9, 28]. Single‐cell transcriptomics further revealed that tumors with robust chemokine signaling and T‐cell infiltration responded better to immunotherapy, whereas immune‐excluded phenotypes were associated with poor efficacy [10, 11]. Collectively, these data support the concept that a “hot” TME enriched in CXCL9/10 signaling and T‐cell infiltration is a critical determinant of responsiveness to immune checkpoint blockade in HCC [7, 8, 9, 10, 11, 28].

In our cohort, high HAMP expression was observed alongside features of immune activation, including increased CD8+ infiltration. We interpret this as part of an inflamed TME conducive to ATZ + BeV efficacy, rather than as a sole causal determinant. HAMP may represent a molecular link connecting iron metabolism, STAT3 signaling, immune infiltration, and immune checkpoint molecule expression [8, 9, 10, 11, 17, 18, 20, 21, 22, 23]. Its expression could therefore contribute to a composite biomarker panel, alongside T‐effector signatures, PD‐L1, and CD8+ density, to better predict therapeutic response [9, 11, 22, 28]. Future studies using spatial transcriptomics and single‐cell analyses are warranted to define the spatial relationship between HAMP expression and immune cell localization and to characterize immune subsets associated with therapeutic benefit [10, 11, 28].

Although our cohort is small and derived from specimens resected after ATZ + BeV, these data are relevant to the preoperative setting. If validated, high tumoral HAMP together with high CD8+ TIL density may help identify patients more likely to achieve meaningful pathological response and proceed to resection after anti–PD‐L1/anti‐VEGF‐based conversion therapy. These signals could inform patient selection and surgical timing within multidisciplinary care.

This study has some limitations. The small sample size limits the generalizability of the findings and warrants cautious interpretation. Moreover, one institutional case was excluded due to RNA quality concerns, which might have affected the observed gene expression profiles and introduced potential confounding. Future investigations should validate the reproducibility and clinical relevance of the candidate genes and pathways identified here using larger, multi‐institutional cohorts with more diverse patient populations. Such efforts, coupled with advanced analytical approaches, will be essential to further elucidate the molecular mechanisms underlying the response to ATZ + BeV therapy in HCC. Owing to the unavailability of pretreatment tissue and reliance on post‐treatment resections, we could not distinguish between the treatment‐induced and pre‐existing expression. Accordingly, the RNA‐Seq results should be interpreted as exploratory rather than predictive. Small‐sample adjusted and stratified analyses suggest that the associations of HAMP and CD8 with response are not entirely attributable to baseline tumor size or serum markers. Confirmation in larger cohorts is required.

## 5 Conclusions

This study suggested that elevated HAMP expression co‐occurred with an inflamed TME marked by increased CD8+ T‐cell infiltration in patients responding to ATZ + BeV therapy. While these findings highlight an association between HAMP‐related biology and therapeutic benefits, they do not establish causality or clarify its relationship with PD‐L1 or STAT3 signaling. HAMP should therefore be considered a potential component of a composite biomarker, the clinical relevance of which requires validation in larger, multi‐institutional, prospective studies incorporating spatial and single‐cell analyses.

## Author Contributions

**Shun Nakamura:** conceptualization, methodology, data curation, investigation, formal analysis, validation, visualization, project administration, writing – original draft, resources. **Yuki Murakami:** data curation, investigation, validation, formal analysis, visualization. **Yusuke Oshima:** conceptualization, methodology, investigation, validation, formal analysis, supervision, project administration, data curation, writing – review and editing. **Takashi Masuda:** conceptualization, methodology, investigation, validation, formal analysis, supervision, project administration, resources, data curation, writing – review and editing. **Wataru Miyoshino:** writing – review and editing. **Hiroomi Takayama:** writing – review and editing, resources. **Yoko Kawano:** writing – review and editing. **Teijiro Hirashita:** writing – review and editing, resources. **Yuichi Endo:** writing – review and editing, resources, project administration, supervision. **Masafumi Inomata:** supervision, project administration, writing – review and editing.

## Funding

This research did not receive external funding. Library preparation and sequencing were funded by institutional/departmental resources at Oita University. The funders had no role in study design; data collection, analysis, or interpretation; writing of the report; or the decision to submit the article for publication.

## Ethics Statement

Approval of the research protocol by an Institutional Reviewer Board: The protocol for this research study has been approved by the Ethics Committee of Oita University (Approval No. 3157) and was conducted in accordance with the principles of the Declaration of Helsinki.

## Consent

Given the retrospective design and minimal risk, the requirement for written informed consent was waived by the Ethics Committee of Oita University (Approval No. 3157). In lieu of obtaining individual consent, an opt‐out approach was implemented by disclosing study information on the institutional website and providing patients with the opportunity to decline participation.

## Conflicts of Interest

Dr. Masafumi Inomata serves on the Editorial Board of Annals of Gastroenterological Surgery. He had no role in the editorial evaluation, peer review, or decision‐making for this manuscript. All other authors declare no conflicts of interest. RNA‐sequencing by Rhelixa Co. Ltd. was performed on a fee‐for‐service basis; the company had no role in the study design, data analysis, interpretation, manuscript preparation, or the decision to publish.

## Supporting information


**Figure S1:** Public RNA‐Seq data. (a) Heatmap of differentially expressed genes (DEGs) comparing responders (R1–R6) and non‐responders (NR1–NR4). (b) MA plot showing significantly upregulated (red) and downregulated (green) genes (adjusted *p* < 0.05, |log_2_ fold change| ≥ 1.5). (c) Volcano plot showing log_2_ fold change versus –log_10_ adjusted *p*‐values.

**Figure S2:** Institutional RNA‐Seq data. (a) Heatmap comparing treated (Atz_Human1–3) and untreated (none_Human1–3) samples; Atz_Human2 was excluded from downstream DEG and enrichment analyses due to RNA quality issues. (b) MA plot showing significantly upregulated (red) and downregulated (green) genes in treated tumors. (c) Volcano plot of log_2_ fold change versus –log_10_ adjusted *p*‐values.

## Data Availability

The datasets generated and/or analyzed during the current study are available from the corresponding author on reasonable request. Processed RNA‐Seq count matrices and code used for downstream analyses will be shared upon request. Public RNA‐Seq data were retrieved from GEO (accession GSE279750).
