# Supplementary figures and images for: Association of HAMP Expression and CD8+ T‐Cell Infiltration With Atezolizumab–Bevacizumab Response in Hepatocellular Carcinoma
Source: Ann Gastroenterol Surg. 2026 Jan 4;10(3):871–82. doi: 10.1002/ags3.70158 (PMC13178277; doi:10.1002/ags3.70158)

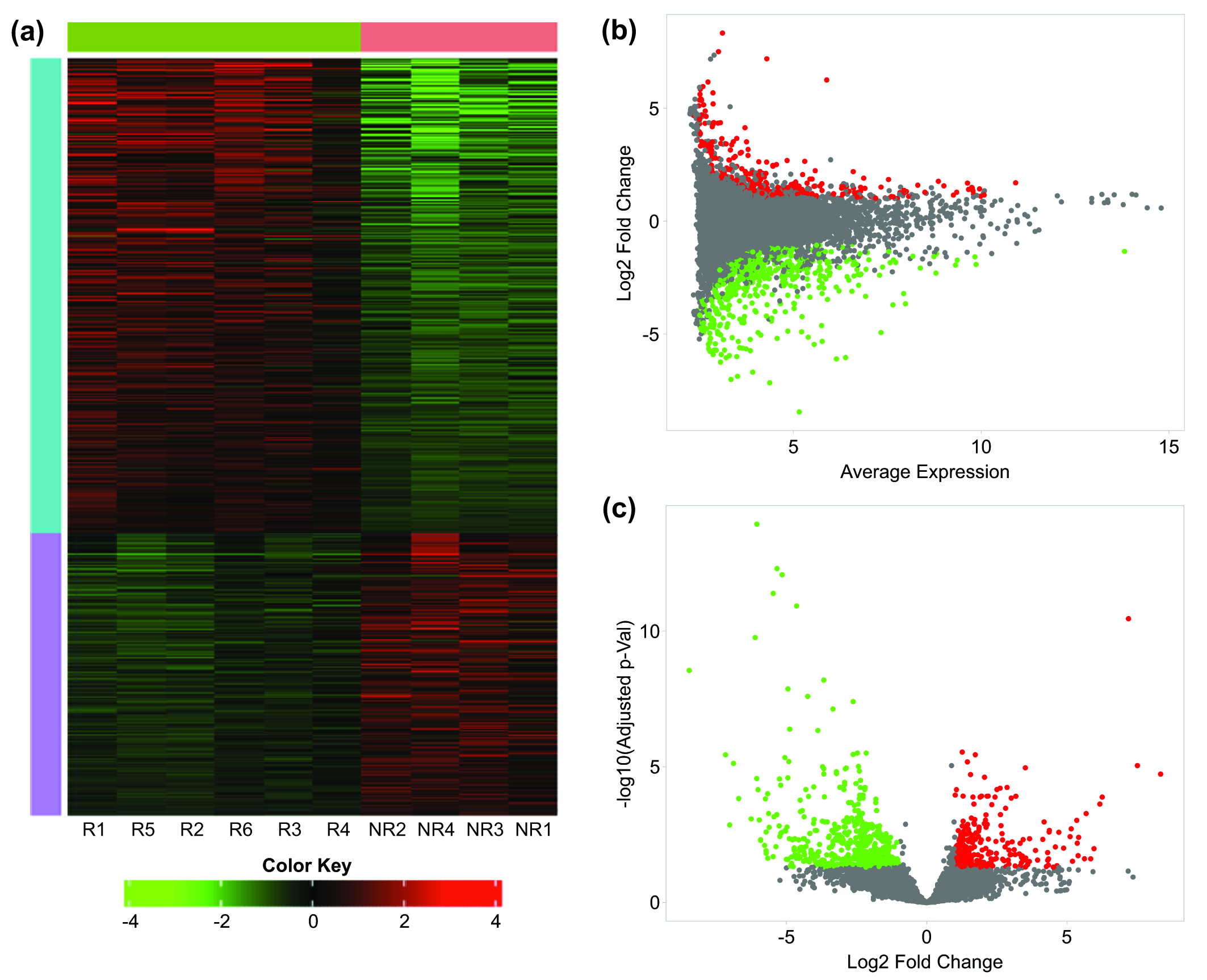

Supplement: Supplementary file 1 — Figure S1: Public RNA‐Seq data. (a) Heatmap of differentially expressed genes (DEGs) comparing responders (R1–R6) and non‐responders (NR1–NR4). (b) MA plot showing significantly upregulated (red) and downregulated (green) genes (adjusted p < 0.05, |log2 fold change| ≥ 1.5). (c) Volcano plot showing log2 fold change versus –log10 adjusted p‐values. [file AGS3-10-871-s001.tif]

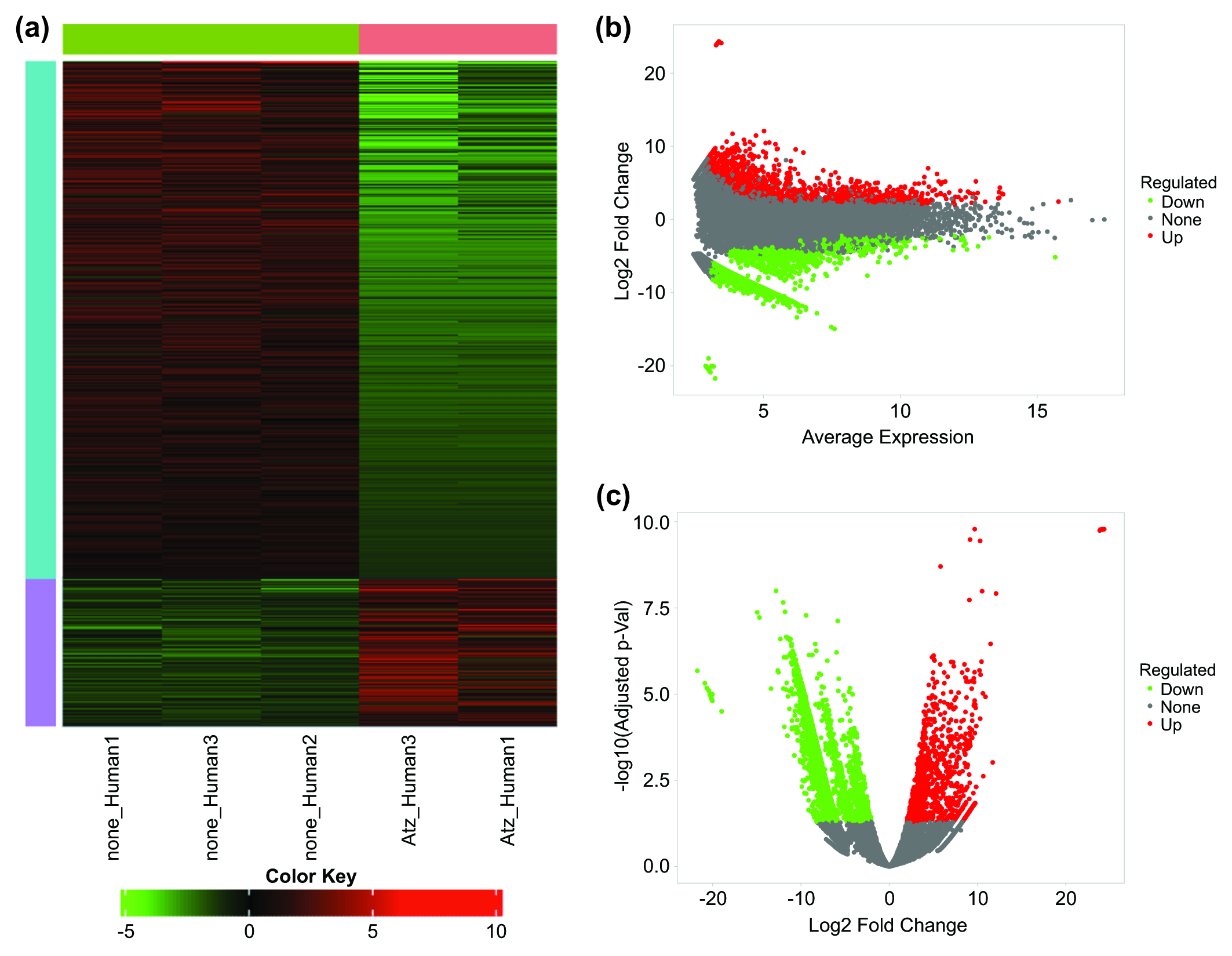

Supplement: Supplementary file 2 — Figure S2: Institutional RNA‐Seq data. (a) Heatmap comparing treated (Atz_Human1–3) and untreated (none_Human1–3) samples; Atz_Human2 was excluded from downstream DEG and enrichment analyses due to RNA quality issues. (b) MA plot showing significantly upregulated (red) and downregulated (green) genes in treated tumors. (c) Volcano plot of log2 fold change versus –log10 adjusted p‐values. [file AGS3-10-871-s002.tif]
